# *Eucalyptus obliqua* seedling growth in organic vs. mineral soil horizons

**DOI:** 10.3389/fpls.2015.00097

**Published:** 2015-02-20

**Authors:** Karen M. Barry, David P. Janos, Scott Nichols, David M. J. S. Bowman

**Affiliations:** ^1^Tasmanian Institute of Agriculture and School of Land and Food, University of TasmaniaHobart, TAS, Australia; ^2^Department of Biology, University of MiamiCoral Gables, FL, USA; ^3^School of Biological Sciences, University of TasmaniaHobart, TAS, Australia

**Keywords:** ashbed effect, mineral nutrition, phosphorus limitation, soil fumigation, ectomycorrhiza

## Abstract

*Eucalyptus obliqua*, the most widespread timber tree in Tasmania, is a pioneer after fire which can eliminate the organic layer of forest soil, exposing the underlying mineral soil. We compared seedling growth, mycorrhiza formation, and mineral nutrient limitation in organic layer vs. mineral soil. We grew *E. obliqua* seedlings separately in pots of organic layer and mineral soil in a glasshouse. Additional treatments of organic soil only, involved fully crossed methyl-bromide fumigation and fertilization. Fertilization comprised chelated iron for 121 days after transplant (DAT) followed by soluble phosphorus. At 357 DAT, whole plant dry weight was three times greater in ambient organic than in mineral soil. In organic soil, fumigation halved ectomycorrhiza abundance and reduced seedling growth at 149 DAT, but by 357 DAT when negative effects of fumigation on seedling growth had disappeared, neither fumigation nor fertilization affected mycorrhiza abundance. Iron fertilization diminished seedling growth, but subsequent phosphorus fertilization improved it. *E. obliqua* seedlings grow much better in organic layer soil than in mineral soil, although phosphorus remains limiting. The prevalent forestry practice of burning to mineral soil after timber harvest exposes a poor growth medium likely only partially compensated by fire-induced mineral soil alterations.

## Introduction

*Eucalyptus obliqua* L'Hér. (Myrtaceae) is a tree species of broad ecological amplitude reflecting diverse ecotypes (Anderson and Ladiges, 1978; Wilkinson, 2008; Bloomfield et al., 2011) in south-eastern Australia including Tasmania. In cool, wet, mountainous areas it forms tall, open forests, sometimes mixed with other eucalypt species such as the closely-related *E. regnans* F. Muell. which tends to predominate at higher elevation, wetter sites than *E. obliqua*. Eucalypt basal areas in these forests can range from 60 to 90 m^2^ ha^−1^ with heights of 40–65 m (Neyland et al., 2012). *E. obliqua* is capable of becoming a giant tree with a reported maximum height of 98.8 m (Tng et al., 2012).

Like other giant eucalypts including *E. regnans* (Dignan et al., 1998; Van Der Meer et al., 1999) and the tropical *E. grandis* W. Hill ex Maiden (Doley, 1978), *E. obliqua* is light-demanding, needing at least 27% of full sun photosynthetically active radiation to maintain sapling intermediate foliage (Alcorn, 2002). Rapid early growth is imperative for seedlings to avoid being overtopped by competing vegetation (Neyland et al., 2009), but eucalypt seedlings often are poor competitors for soil resources needed to achieve the rapid growth of which they are capable (Withers, 1979). Consequently, natural regeneration depends upon high intensity fires that open the canopy, eliminate dense understory vegetation, and bare the soil (Tng et al., 2012). The stands to which intense fires give rise typically represent single cohorts of the fire-intolerant *E. regnans* which is an obligate-seeder with thin bark, but are likely to represent multiple cohorts of *E. obliqua* which has thick fibrous bark and epicormic buds allowing recovery from all but the most severe fires (Turner et al., 2009). With its intolerance of shade and belowground competition, and a capacity for dense recruitment (Ashton, 1986) and rapid early growth, *E. obliqua* essentially is a long-persistent, pioneer species in rainforest environments where the primary disturbance is landscape fire (Tng et al., 2012).

Landscape fires have multiple abiotic and biotic effects on soils, known collectively as the “ashbed effect” (Humphreys and Lambert, 1965). The ashbed effect may include direct phosphorus fertilization by ash (Humphreys and Lambert, 1965); increased calcium and magnesium oxides and thereby, increased pH (Attiwill and Leeper, 1987); diminished phosphorus adsorption with dehydration and aggregation of clay (Chambers and Attiwill, 1994); an increased ratio of ammonium to nitrate that may facilitate iron reduction (Mengel, 1994); increased solubility of organic carbon (Raison and McGarity, 1980); diminished soil organic matter, inhibition of organic matter-clay linkages, and promotion of clay eluviation (McIntosh et al., 2005); and reduced allelopathy (Ashton and Willis, 1982). There also may be changes in soil microbiology such as substantial microbe death releasing nitrogen, phosphorus, and manganese (Chambers and Attiwill, 1994) even though culturable microorganisms except for Actinomycetes regain their population sizes within a year (Ashton and Kelliher, 1996); elimination of antagonistic microbes (Florence and Crocker, 1962); disruption of common arbuscular mycorrhizal networks which may have intensified competition with rain forest plants (Janos et al., 2013); and Ascomycetes replacing Basidiomycetes as ectomycorrhizal associates (Warcup, 1991; Launonen et al., 2004).

The complexity of the ashbed effect defies simple attribution of growth stimulation following fire or growth inhibition in unburnt stands. Nevertheless, high intensity fire is widely considered indispensable for regeneration following logging because it mimics natural, stand-replacing fires. Ashbeds are produced by the widely-prevailing forestry practice of “clear-fell, burn, and sow” (Attiwill, 1994). After clear-fell, the highest seedling densities and fastest early growth rates are associated with the hottest burnt seedbeds (Neyland et al., 2009, 2012). In marked contrast, however, Facelli and Kerrigan (1996) found that ash strongly reduced emergence, increased mortality, and led to the lowest *E. obliqua* seedling biomass in a pot experiment. Subsequently, Ladd and Facelli (2008) found that *E. obliqua* seedlings free from competitors in pots and in the field attained greater biomass in the presence than in the absence of litter. Such seeming inconsistencies suggest that how specific attributes of seedbeds affect *E. obliqua* performance needs further elucidation. So, here we briefly review two interrelated aspects of the ashbed effect involving seedling mineral nutrition and mycorrhizas and report an investigation of them.

*E. obliqua* seedlings require elevated fertility to achieve their highest growth rates. With a pot experiment, Attiwill (1962) showed that either adding 30 ppm phosphorus to surface soil from a mature stand of *E. obliqua* or heating the soil to 500°C enhanced seedling growth. Attiwill (1962) showed a 30 ppm nitrate addition had little effect on seedling growth although nitrogen fertilization increased the height of 5-year-old *E. obliqua* saplings in the field (Cromer et al., 1981). Also in the field, phosphorus fertilization increased the growth of *E. regnans* to 45 months, but nitrogen fertilization had no detectable effect (Bennett et al., 1996). In accord with the latter finding, Attiwill and May (2001) concluded that phosphorus was more likely than nitrogen to be limiting to ecological processes such as litter decomposition in *E. regnans* forests. *E. regnans* and *E. obliqua* forests grow on soils conspicuously poor in phosphorus which explains why before litter fall, there is withdrawal and redistribution to biomass of about 70% of foliar phosphorus in these species (Attiwill et al., 1978).

While phosphorus is of greatest importance in the mineral nutrition of *E. obliqua*, iron and manganese have been studied as well. Anderson and Ladiges (1978) compared growth in pots of *E. obliqua* seedlings from three populations on soils ranging in pH from 4.6 to 8.0, and found that those from a tall open-forest on an acidic loam were severely chlorotic with markedly reduced yield when grown on calcareous sands. Addition of an iron chelate could alleviate the lime-chlorosis (Anderson, 1983) as could heating the soil by burning (Anderson and Ladiges, 1982). Lime-chlorosis also can be associated with low foliar concentrations of manganese (Czerniakowski et al., 2006), but *E. obliqua* and *E. regnans* seedlings had higher total dry weights in pots of a sand with low (42 mg kg^−1^) total manganese than in soils with either moderate (301 mg kg^−1^) or high (928 mg kg^−1^) manganese (Hill et al., 2001). Because of the low solubility and mobility of iron in most soils, any factors that inhibit root growth, such as high pH and associated high bicarbonate concentrations which also reduce manganese availability, may be associated with foliar interveinal chlorosis (Parsons and Uren, 2007). At low pH, aluminum and copper toxicities (Foy, 1992) can reduce root growth and thereby interfere with iron and manganese uptake even though low pH may increase the availability of all these elements.

As adults, all species of *Eucalyptus* that have been examined are capable of forming ectomycorrhizas as their primary means of acquiring mineral nutrients, especially in leached soils with accumulations of organic material (Chilvers and Pryor, 1965). Young eucalypts, however, quickly may form arbuscular mycorrhizas that later are succeeded by ectomycorrhizas (Chilvers et al., 1987; Adams et al., 2006). Nevertheless, formation of arbuscular mycorrhizas and an ontogenetic shift to ectomycorrhizas with age probably is not obligatory because *E. regnans* seedlings, for example, may form ectomycorrhizas within three weeks of germination (Launonen et al., 2004). Whether *E. obliqua* seedlings form arbuscular mycorrhizas is uncertain. Warcup (1991) reported “vesicular-arbuscular mycorrhizas” in mixed *E. obliqua* and *E. regnans* forests, but did not specifically attribute them to either host species. Nevertheless, *E. obliqua* unequivocally has been demonstrated to form ectomycorrhizas with several Basidiomycetes and Ascomycetes (Malajczuk et al., 1982; Warcup, 1991).

Ectomycorrhizas generally have been shown to stimulate eucalypt seedling growth, and Pryor (1956a,b) argued that ectomycorrhizas might be obligatorily required by “renantherous” eucalypt species, including *E. obliqua* and *E. regnans*, because of their failure to grow in exotic plantations in the northern hemisphere that may have lacked appropriate fungi. Ashton (1976), however, stated that ectomycorrhizas were not necessary for *E. regnans* seedling survival if mineral nutrients were adequate [moreover, *E. regnans* seedlings can develop copious root hairs when not ectomycorrhizal (Launonen et al., 2004)], and Pryor (1956a,b) did note that species of other sections of *Eucalyptus* could grow in heat-sterilized soil without mycorrhizas. Thus, eucalypts are likely to be facultatively ectomycorrhizal—able to grow without mycorrhizas in sufficiently fertile soil (Janos, 2007)—consistent with their pioneer status (Janos, 1980).

Although ectomycorrhizas have been demonstrated to enhance the growth of *E. regnans* seedlings (Ashton, 1976; Launonen et al., 1999, 2004), we found no reports of the effects of either ectomycorrhizas or arbuscular mycorrhizas on *E. obliqua*. Similarly, the consequences of changes in species composition of the fungi that form ectomycorrhizas in stands of different ages (Gates et al., 2011), soon after fire (Warcup, 1991; Launonen et al., 1999; McMullan-Fisher et al., 2011), or even after air drying of soil (Launonen et al., 2004, 2005) are poorly known for *E. regnans* and apparently unknown for *E. obliqua*.

In this study we investigate the synergistic effects of soil nutrition and mycorrhizas by contrasting *E. obliqua* seedling growth in pots of wet eucalypt forest mineral vs. organic soil layers. We also investigated seedling growth in organic soil subjected to factorial combinations of fumigation and fertilization. We hypothesized that: (1) seedlings would grow better in ambient (i.e., neither fumigated nor fertilized) organic than in ambient mineral soil because of greater mineral nutrient availability; (2) methyl-bromide fumigation of organic soil would retard ectomycorrhiza formation and thereby reduce seedling growth; and (3) chelated iron or phosphorus fertilization of organic soil would increase seedling growth.

## Materials and methods

### Experiment design and soil treatments

Our experiment consisted of five treatments in an unbalanced design. Four of those, applied only to organic layer soil, were a factorial combination of fumigation (two levels: “ambient” = not fumigated vs. fumigated) and fertilization (two levels: none vs. addition of an iron chelate which was discontinued and supplanted by soluble phosphorus addition). The fifth treatment was ambient, non-fertilized mineral soil.

**Figure 1:**
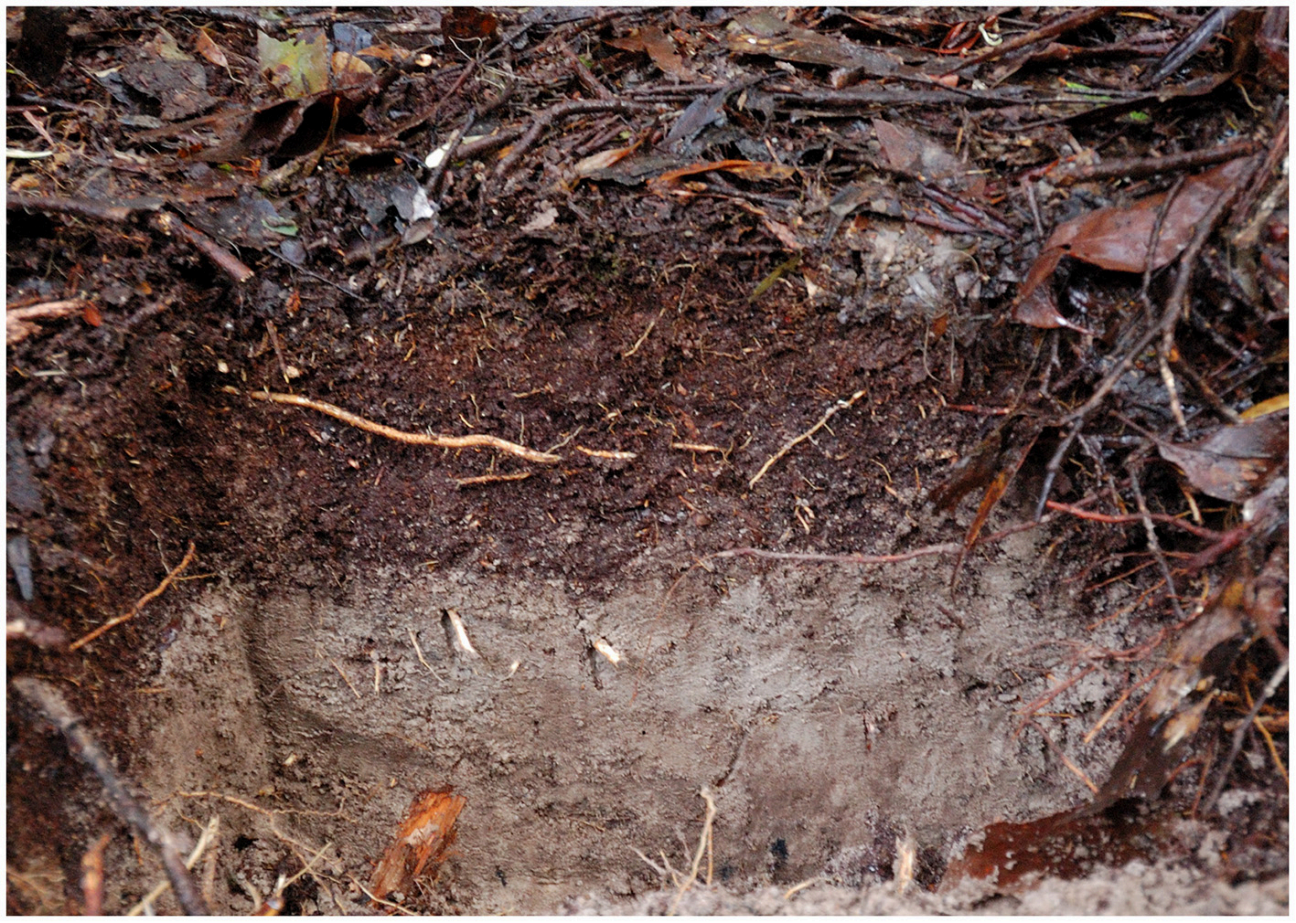
**Partial profile of *Eucalyptus obliqua* forest soil near the sites of soil collection (S 43.126838°, E 146.700472°) along West Picton Road in southern Tasmania**. When collected, the surface litter (visible at the top of the photo) was removed, and the dark brown, organic layer soil was separated from the light gray-brown mineral soil beneath it.

The soil, a humose, dystrophic, Brown Kurosol (McKenzie et al., 2004), was collected on 19 March, 2010, from two locations 1 km apart along West Picton Road in an *E. obliqua* forest in southern Tasmania (43.126838°S, 146.700472°E). Surface litter was removed before the soil was excavated to a depth of about 30 cm. The uppermost, 5–10 cm thick organic layer (A1 horizon) was separated from the underlying, bleached mineral soil (A2j horizon) at their abrupt boundary (Figure 1). The soil was typical for the region (Laffan, 2001). Soil from both locations was combined separately for each layer, thoroughly mixed, and then was allowed to air dry indoors for 8 weeks. After approximately 1300 cm^3^ of soil was dispensed to 20 plastic pots (120 mm diameter × 140 mm deep) for each treatment, one-half of the pots containing organic soil were treated with methyl bromide gas at a rate of 48 g m^−3^ for 24 h at 21°C (Tasmanian Ports Quarantine Facility, Hobart). The mineral soil was neither fumigated nor subsequently fertilized.

In an effort to reintroduce the native soil bacterial and saprotrophic fungus microflora to fumigated organic soil while excluding arbuscular mycorrhizal fungi and perhaps excluding some ectomycorrhizal fungi, 50 mL of a microbial filtrate was added to each pot containing organic, fumigated soil before seedling transplant. An equal volume of distilled water was added to each non-fumigated pot. We prepared the microbial filtrate by soaking ambient organic layer soil in distilled water overnight (1:1 soil:water), and then vacuum filtering the decanted water through three layers of Whatman No. 1 filter paper.

Because we were concerned that iron uptake from the organic soil might be insufficient, we fertilized half of the pots containing organic soil with chelated iron, 15.832 g L^−1^ “Sequestrene 138” (Sodium ferric ethylenediamine bis 2-hydroxyphenylacetate). Beginning on 2 June, 2010, 50 mL per pot (= 47.5 mg Fe) was added weekly for 14 weeks, half in the morning and half in the evening of the same day, after watering at both times. Plants not fertilized, similarly were given 50 mL of distilled water. At 121 days after transplant (DAT), we discontinued iron fertilization because it negatively affected seedling growth. To compensate for the added iron possibly having diminished phosphorus availability (Yuan and Lavkulich, 1994), on 24 October, 2010 we began weekly addition (one-half in both morning and evening) of 50 mL per pot (= 39.0 mg P) of 3.475 g L^−1^ sodium dihydrogen phosphate monohydrate. We continued phosphorus fertilization until 27 February, 2011, thereby having added 18 doses in total.

### Seedling growth, measurement, and harvest

We collected *E. obliqua* seeds from four parent trees approximately 1 km apart along West Picton Road, near where we had collected soil. The seeds of all four trees were pooled, mixed, and surface-disinfested by 5 min immersion in 2% sodium hypochlorite. Seeds were germinated in trays of a vermiculite-based germination mix which had been autoclaved (121°C, 1.27 kg cm^−2^) for 1 h, rested for 24 h, and then autoclaved again for 1 h. Seeds were allowed to germinate for up to 120 days after sowing before transplant. To begin the experiment, we carefully removed seedlings from the germination trays and rank-ordered them by size while keeping their bare roots moist. In order, we transplanted one seedling per pot to one pot of each of the five treatments in continuing rotation until all 20 pots of each treatment thereby had received seedlings with similar ranges of initial sizes. Any seedlings that died were replaced during the 14 d between transplant and the initial measurement on 2 June, 2010.

Plants were grown in a controlled environment glasshouse in which the temperature was maintained at 20°C day and 10°C overnight with supplementary lighting used to maintain a12/12 h day/night cycle. Positions of all treatments' pots were fully randomized, and the pots were spaced 30–40 cm apart on glasshouse benches. Pot positions were changed weekly. Plants were watered by an automated spray mist system which operated for 10 min at 7:00 h and 10 min at 16:00 h. No pest control was required during the experiment.

Beginning 14 DAT, and following at varied intervals thereafter until the final harvest at 357 DAT, we measured plant height from the soil to the end of highest petiole and, with digital calipers, stem diameter 1 cm above the cotyledons. We counted the number of leaves (not including cotyledons) and measured the length of the longest leaf from tip to base (i.e., excluding the petiole).

In order to assess mycorrhizas with the best chance of detecting any colonization that might have taken place before iron fertilization was switched to phosphorus fertilization, the three visibly largest plants of each treatment were harvested at 149 DAT. All surviving plants among the 17 remaining per treatment were harvested at 357 DAT, almost a full year after transplant. We concluded the experiment at 357 DAT in order to pre-empt pot limitation; the plants in organic soil continued to increase in height, stem diameter, and number of leaves until that time.

At both harvests, shoots were cut at the soil surface and the blades of all leaves were separated from petioles and stems. The three largest leaves from each plant were scanned immediately with a desktop scanner, and their area was determined with Image J (http://imagej.nih.gov/ij/index.html). All leaves, stems, and petioles were dried to constant weight at 40°C in a fan-forced oven, with scanned leaves dried separately to enable calculation of leaf specific area. Root systems were extracted from pots over a 212 μm sieve under a gentle stream of water before storage in 60% ethanol in anticipation of sub-sampling for mycorrhiza assessment. After sub-sampling, root systems were oven dried at 40°C to constant weight. We determined the dry weights of all harvested plant parts separately.

We ground the dried leaves of each plant in a ball grinder (Retsch MM200) for 2 min before sending the ground tissue to CSBP Limited, Soil and Plant Analysis Laboratory, Western Australia for analysis. Foliar N, P and other elements were determined after complete acid digestion of the plant material followed by inductively coupled plasma–atomic emission spectrometry (ICP-AES).

At the final harvest, we used a 0.75 cm inside-diameter sharpened copper tube to extract a soil core from each pot to a depth of 5.0 cm. The cores were dried separately to constant weight before weighing to approximate bulk density. After extracting root systems from each pot, we collected ca. 100 g of soil from each, pooled and thoroughly mixed it by treatment before air-drying for 2 week. For each treatment, a sub-sample of 500 g soil was analyzed by CSBP Limited. Ammonium and nitrate were extracted in KCl and quantified with a Lachat Flow Injection Analyser. P and K (Colwell) were extracted in sodium bicarbonate, Cu, Fe, Mn, and Zn in DTPA, and both extracts analyzed by atomic absorption spectrometry. Al, Ca, Mg, K, and Na were extracted in ammonium chloride and analyzed with ICP-AES. Soil pH and electrical conductivity were determined in a 1:5 soil:water suspension, and pH was determined also in a calcium chloride suspension.

On 9 November, 2014, in order to approximate “beginning” soil attributes, we collected additional soil samples from the same two locations as previously. We manually removed woody debris and roots, then composited, thoroughly mixed, and oven-dried the organic and mineral soil layers separately before having them analyzed by CSBP Limited.

### Mycorrhiza assessment

To assess ectomycorrhizal colonization, we sub-sampled the preserved roots of all three early harvest plants and of six randomly-selected final harvest plants per treatment. The preserved roots were blotted dry and weighed before a sub-sample was removed, weighed, and the weight deducted from that of the sample. The weight ratio before and after drying was used to estimate the dry weights of sub-samples so that total root weights could be calculated. From the sub-samples, intact root tips were discerned with a dissecting microscope (50 ×) and placed on microscope slides without staining. With a compound microscope at 100–400 × magnification, we examined 50 root tips per plant for ectomycorrhizal mantles, extraradical hyphae, and clamp connections. We attempted to distinguish morphotypes of ectomycorrhizas based upon color, mantle morphology, and extraradical hypha characteristics.

### Statistical analyses

Because of our unbalanced experiment design, our general approach to statistical analyses was to separately compare seedling performance in mineral soil to that in only ambient organic soil (i.e., both not-treated soils) by One-Way analysis of variance (ANOVA), and then to compare the effects of fumigation and fertilization on organic soil alone by Two-Way, factorial ANOVA. Levene's test was used to check homogeneity of variances, and except where otherwise noted, analyses of multiple response variables were Bonferroni-corrected for the number of variables analyzed. All statistical analyses were conducted with Statistix 10.0 except for repeated-measures analyses which used REML estimation in JMP Pro 10.0.0.

Levene's test showed soil bulk densities to be heteroscedastic, but a log_10_ transformation homogenized variances so that all five treatments could be compared in a One-Way ANOVA. Because we used single, composited samples to represent each treatment for other soil attributes, we performed separate, Two-Way, factorial ANOVAs for each attribute for only the four organic soil treatments by using the interaction term as an error estimate. Because of the weakness of these analyses, we did not Bonferroni-correct for the number of attributes analyzed, and we accepted *P* ≤ 0.09 as suggestive of differences among treatment means. Moreover, because they represented collections at different times, we did not statistically compare “beginning” soil attributes to those of soils taken from pots at the end of the experiment.

Percentage ectomycorrhizal root tips were analyzed separately for each harvest because we intentionally had biased the initial harvest to the largest plants of each treatment. We analyzed arcsine-square-root-transformed percentages for each harvest by One-Way ANOVA because the variances were homogenous among all five treatments. We used Pearson's correlation coefficients to examine associations between whole plant dry weight and percentage ectomycorrhizal root tips.

Using our general approach, we examined four morphological response variables (height, longest leaf length, number of leaves, and stem diameter) with repeated-measures ANOVAs and Bonferroni-corrected probabilities (*P* ≤ 0.05/4 = 0.0125). For the repeated-measures analyses, however, we separated the time intervals during which we fertilized with Fe (0–121 DAT) and P (121–357 DAT). Because the three largest plants of each treatment were harvested at 149 DAT, we excluded those 15 plants from the repeated-measures analyses for both intervals. For the second time interval, all individual plant measurements were relativized by deduction of their values at the beginning of the interval in order to reflect changes in size during the interval. In comparing the mineral vs. ambient organic soil, leaf length was log_10_-transformed for the first interval, but no parametric transformation homogenized diameter variances, so diameter was rank-transformed. For the second interval, height change only was log_10_-transformed. Number of leaves change was marginally heteroscedastic according to Levene's test (*P* = 0.0458), so was not transformed. All organic soil treatments were homoscedastic for both intervals.

When we considered the final harvest dry weights of leaves, stems, roots, total plant, and root-to-shoot ratios for mineral vs. ambient organic soil, leaf and stem weights were heteroscedastic, so we used Welch's test for mean differences (Welch, 1951) for all five comparisons with a Bonferroni-corrected probability of *P* ≤ 0.0100. We also used Welch's test to compare mean leaf specific areas for these two treatments. When only organic soil treatments were compared, all final harvest response variables were homoscedastic, and so we used Two-Way ANOVAs with effects Bonferroni-corrected as for the mineral vs. ambient organic soil treatment comparisons followed by Tukey's honestly significant difference tests to separate means.

Mineral nutrient concentrations in leaf tissue of plants grown in mineral soil vs. ambient organic soil were heteroscedastic for Mg, Mn, Na, and N:P (Levene's test *P* ≤ 0.0245), so we compared all elements by using Welch's test with a Bonferroni-corrected probability of *P* ≤ 0.0038 (12 elements and one element ratio). Mineral nutrient contents (= concentration × leaf dry weight) were homoscedastic only for Fe and Cu, so we compared all elements by using Welch's test with a Bonferroni-corrected probability of *P* ≤ 0.0042. For plants in all organic soil treatments, P and Fe concentrations were heteroscedastic (Levene's test *P* ≤ 0.0004) as were P, Fe, and Zn contents (Levene's test *P* ≤ 0.0326), but in all those instances log_10_ transformations resulted in homoscdasticity. We compared foliar element concentrations and contents among plants grown in all organic soil treatments by Two-Way ANOVAs with Bonferroni corrections followed by Tukey's tests.

## Results

### Soil fertility

Non-burnt mineral soil is far denser and generally less fertile than ambient organic soil. Mineral soil bulk density significantly exceeded [*F*_(4, 75)_ = 480.83, *P* < 0.0001] by 10-fold the grand mean of all organic soil treatments (which did not differ; Table 1). Ambient organic soil had almost four times more available (Colwell) P than mineral soil and had conspicuously higher concentrations of most other mineral nutrients including K, Mn, Zn, Cu, Ca, and Mg. Only Fe and Al were higher in the mineral soil than in the organic soil (Table 1). Field soils assumed to represent “beginning” attributes showed the same pattern of only Fe and Al being higher in mineral than organic soil (Table 1). Decreased mineral nutrient concentrations in mineral and ambient organic soils taken from pots at harvest vs. the field were greater than 50% on average across both soils only for Fe, Colwell K, and exchangeable K.

**Table 1:** **Physical and chemical attributes of field-collected (Field) mineral and organic (Org.) soil layers, and of those layers and treated organic soil after 357 days of *Eucalyptus obliqua* seedling growth in pots**.

**Attribute^a^ Units**	**Field mineral**	**Mineral**	**Field Org. Org**.	**Org., Amb.^b^**	**Org., Amb., Fert**.	**Org., Fum**.	**Org., Fum., Fert**.
Bulk density^c^ g cm^−3^	ND	0.93 a (0.012)	ND	0.09 b (0.012)	0.08 b (0.013)	0.09 b (0.013)	0.10 b (0.013)
Conductivity dS m^−1^	0.067	0.047	0.207	0.193 a^d^	0.272 a	0.177 a	0.176 a
pH (CaCl_2_)	3.75	3.5	2.3	3.3 a	3.3 a	3.2 a	3.3 a
pH (H_2_O)	4.25	4.4	3.4	4.4 a	4.3 a	4.2 a	4.3 a
Ammonium nitrogen mg kg^−1^	4	6	52	9 a	362 a	244 a	213 a
Nitrate nitrogen mg kg^−1^	<1	<1	<1	<1	1	<1	<1
Phosphorus (Colwell) mg kg^−1^	<2	10	21	38 b	**287 a**	69 b	**299 a**
Potassium (Colwell) mg kg^−1^	70	41	435	100 a	163 a	129 a	153 a
Iron (DTPA) mg kg^−1^	290.33	152.69	105.24	49.63 b	**392.92 a**	73.59 b	**362.40 a**
Manganese (DTPA) mg kg^−1^	0.36	0.96	11.24	1.87 b	**8.28 a**	3.76 b	**12.18 a**
Zinc (DTPA) mg Kg^−1^	0.37	2.26	5.10	21.10 a	23.53 a	23.53 a	25.33 a
Copper (DTPA) mg kg^−1^	0.51	1.16	2.36	15.32 a	8.35 a	14.04 a	9.87 a
Exchangeable Aluminum meq 100 g^−1^	3.209	1.539	0.84	0.718 a	0.714 a	0.871 a	1.008 a
Exchangeable Calcium meq 100 g^−1^	0.17	0.76	8.05	11.86 a	14.31 a	14.59 a	13.93 a
Exchangeable Magnesium meq 100 g^−1^	0.23	0.42	6.87	4.64 a	5.28 a	6.27 a	5.99 a
Exchangeable Potassium meq 100 g^−1^	0.15	0.10	1.13	0.26 a	0.42 a	0.33 a	0.39 a
Exchangeable Sodium meq 100 g^−1^	0.08	0.05	0.85	0.53 b	**1.71 a**	0.70 b	**1.59 a**

a *Field soil attributes are means of two subsamples of thoroughly mixed organic and mineral soil layers from two collection sites. All attributes of soils from pots except bulk density reflect single measurements of pooled samples from all pots within a treatment*.

b *Amb., ambient soil (i.e., not fumigated); Fum., fumigated with methyl bromide gas; Fert., fertilized with chelated iron followed by phosphate (see Materials and Methods)*.

c *ND, not determined. Treatment means (± SE); n = 17 per treatment. Means followed by the same lowercase letter do not differ significantly at P ≤ 0.05 by Tukey's honestly significant difference test (after log_10_ transformation of the data)*.

d *Within an attribute other than bulk density, among organic soil treatments only from pots, values followed by the same lowercase letter do not differ significantly at P ≤ 0.09 by two-way analysis of variance using the interaction term as the error estimate. Significantly elevated values are shown in bold. Nitrate nitrogen was not tested*.

As expected, Fe followed by P fertilization of organic soil persistently and significantly elevated both DTPA-extractable Fe [*F*_(1, 1)_ = 134.62, *P* = 0.0547; Table 1] and available P [*F*_(1, 1)_ = 635.57, *P* = 0.0252]. Extractable Mn [*F*_(1, 1)_ = 54.44, *P* = 0.0858] and exchangeable Na [*F*_(1, 1)_ = 50.95, *P* = 0.0886] also were elevated in consequence of fertilization. No other soil attributes were influenced significantly by either fertilization or fumigation (all *P* ≥ 0.094). Nevertheless, it merits note that vs. ambient organic soil, fumigation (as well as fertilization) greatly elevated ammonium N (although we could not support this statistically because of using the interaction to estimate error). Phosphorus, Fe, and Mn also may have been somewhat elevated by fumigation (Table 1).

### Mycorrhizal colonization

**Figure 2:**
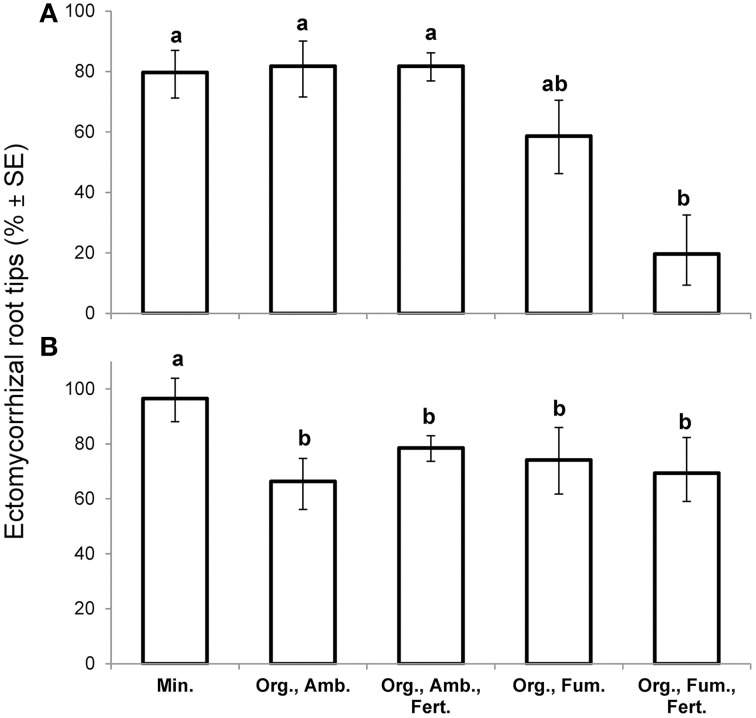
**Mean percentage ectomycorrhizal root tips (% ± SE) of *Eucalyptus obliqua* seedlings at the early harvest, 149 days after transplant (A), *n* = 3 largest plants, and at the final harvest, 357 days after transplant (B), *n* = 6 randomly selected plants**. Standard error bars are not symmetric around means because they are back-transforms of arcsine-square-root-transformed percentages. Within each panel, bars topped by the same lowercase letter do not differ significantly at *P* ≤ 0.05 by Tukey's honestly significant difference test. Min., mineral soil; Org., organic soil; Amb., ambient soil (i.e., not fumigated); Fum., fumigated with methyl bromide gas; Fert., fertilized with chelated iron followed by phosphate (see Materials and Methods).

At the first harvest at 149 DAT, in spite of having collected the largest plants in order to maximize the likelihood of finding any ectomycorrhizas, fumigation reduced the percentage ectomycorrhizal root tips [*F*_(4,10)_ = 6.38, *P* = 0.0081] from 81% in both ambient organic soil treatments to a mean of 40% in the fumigated organic soils, but iron fertilization had no effect (Figure 2A). Ectomycorrhiza percentage in mineral soil did not differ from that in ambient organic soil. Over all treatments, ectomycorrhiza percentage was not significantly associated with whole plant dry weight (n = 15, r = 0.18, *P* = 0.5237) at the first harvest.

At the final harvest, neither fumigation nor fertilization of organic soil affected percentage ectomycorrhizal root tips for which the overall mean was 71%. Nevertheless, 95% ectomycorrhizal root tips in mineral soil exceeded all organic soil treatments [*F*_(4,25)_ = 9.26, *P* = 0.0001; Figure 2B]. Of three common ectomycorrhizal morphotypes that we distinguished, one with a black mantle predominated in mineral soil where it represented 94% of all ectomycorrhizas observed, but it represented only 7.5% of ectomycorrhizas in organic soil. In organic soil, two brown morphotypes were nearly equal in abundance to one another. Clamp connections were twice as common (24.5 vs. 12.2%) on extra-radical hyphae of the morphotypes in organic soil as on the morphotype in mineral soil. There was no significant association between ectomycorrhiza percentage and whole plant dry weight for either plants in mineral soil (*n* = 6, *r* = −0.13, *P* = 0.8038) or those in non-fertilized organic soil (*n* = 12, *r* = −0.23, *P* = 0.4782), but there was a significant, strong positive association in fertilized organic soil (*n* = 12, *r* = 0.69, *P* = 0.0128).

### Plant survival and growth

Among the 17 plants remaining per treatment after the first harvest, survival was high. Only two seedlings died in the fumigated, fertilized organic soil treatment and one in the ambient, fertilized organic soil treatment by 357 DAT.

**Figure 3:**
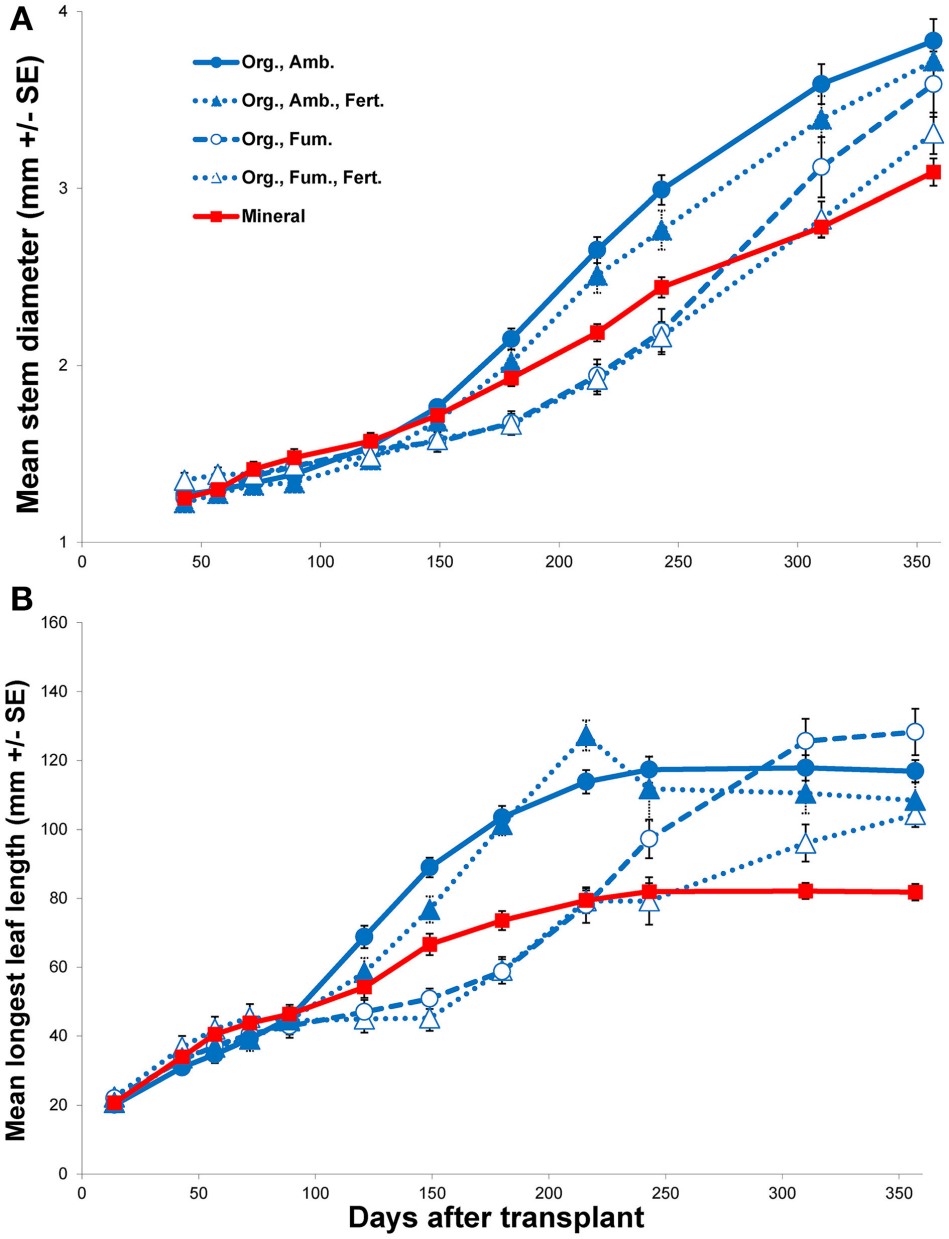
**Mean stem diameter (mm ± SE; A) and mean longest leaf length (mm ± SE; B) vs. days after transplant of *Eucalyptus obliqua* seedlings**. Seedlings were transplanted to mineral soil (solid red line, filled squares; “Mineral”) or organic layer soil (blue lines; “Org.”) that was either fumigated (dashed line, open circles; “Fum.”) or not (solid line, filled circles; “Amb.”) and/or fertilized (dotted lines, filled triangles; “Amb., Fert.;” or dotted lines, open triangles; “Fum., Fert.”). The fertilization regime began with chelated iron, but was changed to phosphate 150 days after transplant. All first harvest plants were excluded.

In spite of all *E. obliqua* seedlings in mineral soil surviving throughout the experiment, their growth eventually became the slowest among all treatments (Figure 3 and Figure S1). When compared to ambient organic soil alone, although no main effects of soil were significant (all *P* > 0.4870), there were significant soil × DAT interactions over the 121 day first interval for height [*F*_(5, 160)_ = 5.92, *P* < 0.0001], longest leaf length [*F*_(5, 160)_ = 6.69, *P* < 0.0001], and number of leaves [*F*_(5, 160)_ = 8.41, *P* < 0.0001], but not for stem diameter [*F*_(4, 128)_ = 2.39, *P* = 0.0541]. During the 236 day second interval, the changes in all four variables showed both significant main effects of soil (all *P* < 0.0001) and significant interactions with time (all *P* < 0.0001). For both the first and second intervals, for those variables that differed significantly, seedling growth in ambient organic soil exceeded that in mineral soil. Only mean stem diameter continued to increase throughout the experiment in all treatments, but the increase was slowest in mineral soil (Figure 3A). In contrast, mean longest leaf length tended toward asymptotes in all treatments, among which the asymptote was least in mineral soil (Figure 3B). Mean seedling height and number of leaves both continued to increase substantially for the entire experiment in organic soil, but plateaued in mineral soil (Figure S1).

When we compared only the organic soil treatments during the first interval (Fe addition), as for the mineral vs. ambient organic soil comparison neither the main effects nor their interactions significantly affected any morphological parameter (all *P* ≥ 0.1274). Fumigation × DAT interactions were significant, however, for height [*F*_(5, 310)_ = 9.70, *P* < 0.0001], longest leaf length [*F*_(5, 310)_ = 25.74, *P* < 0.0001], and number of leaves [*F*_(5, 310)_ = 16.61, *P* < 0.0001], but not for stem diameter [*F*_(4, 248)_ = 1.96, *P* = 0.1016]. Fertilization × DAT interactions were significant for all variables (all *P* = 0.0060), but no fumigation × fertilization × DAT interaction was significant after Bonferroni correction (all *P* ≥ 0.0246). By the end of the first interval for the significant morphological response variables, plants in ambient organic soil exceeded those in fumigated soil whether fertilized or not. When fertilized with Fe, plant growth was slowed, especially in ambient soil. Nevertheless, the negative effects of fumigation (on average, an 18% reduction in height, a 28% reduction in longest leaf length, and a 31% reduction in number of leaves) were greater than those of fertilization (on average, a 16% reduction in height, a 10% reduction in longest leaf length, and a 17% reduction in number of leaves).

**Figure 4:**
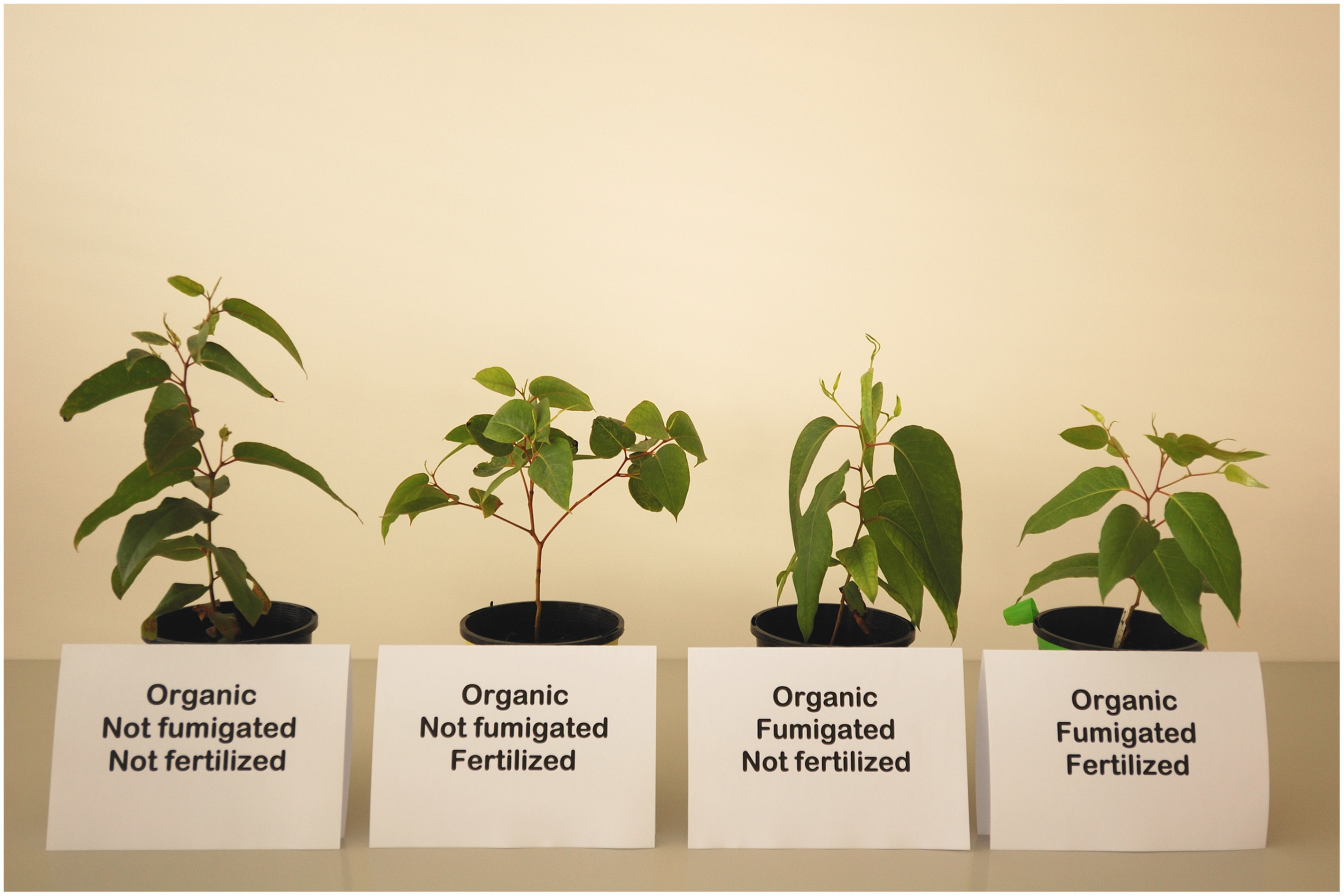
**Approximately “average” *Eucalyptus obliqua* seedlings 357 days after transplant to organic layer forest soil**. Soil was either fumigated with methyl bromide gas or not, and fertilized with chelated iron followed by phosphate (see Materials and Methods) or not. Whether in ambient (not fumigated) or fumigated soil, the fertilized plants were more branched than non-fertilized plants.

For only organic soil during the second interval, there were no significant effects of fertilization (P addition) or the fumigation × fertilization interaction on the change in any morphological variable after Bonferroni correction (all *P* ≥ 0.0280). In contrast, there were significant main effects of fumigation on height change [*F*_(1, 62.04)_ = 19.86, *P* < 0.0001] and stem diameter change [*F*_(1, 62.01)_ = 43.25, *P* < 0.0001], but not on longest leaf length change [*F*_(1, 62.26)_ = 2.99, *P* = 0.0887] or number of leaves change [*F*_(1, 61.78)_ = 0.024, *P* = 0.8771]. As for the first interval, no fumigation × fertilization × DAT interaction was significant after Bonferroni correction (all *P* ≥ 0.0112), but the fumigation × DAT interaction was significant for height change, longest leaf length change, and stem diameter change (all *P* < 0.0001). The fertilization × DAT interaction was significant after Bonferroni correction only for longest leaf length change [*F*_(6, 367.6)_ = 7.52, *P* < 0.0001], but not for height change [*F*_(6, 370.2)_ = 2.53, *P* = 0.0204], number of leaves change [*F*_(6, 370)_ = 1.17, *P* = 0.3197] or stem diameter change [*F*_(6, 370.1)_ = 1.93, *P* < 0.0750]. The initial negative effect of organic soil fumigation on growth generally persisted well into the second interval, although by the end of the interval the growth rates of plants in fumigated organic soil accelerated and differences among treatments in mean plant sizes for all response variables differed little by the final harvest (Figure 3 and Figure S1). The significant fertilization × DAT interaction for longest leaf length change principally reflected a negative effect of P fertilization in fumigated organic soil, but no such effect was detected for any other morphological response variable. Over the entire experiment, the plants in ambient organic soil tended to show the best growth of all treatments. Although not quantified, fertilized plants in organic soil tended to branch more than those not fertilized (Figure 4).

### Plant dry weights and leaf tissue analyses

**Figure 5:**
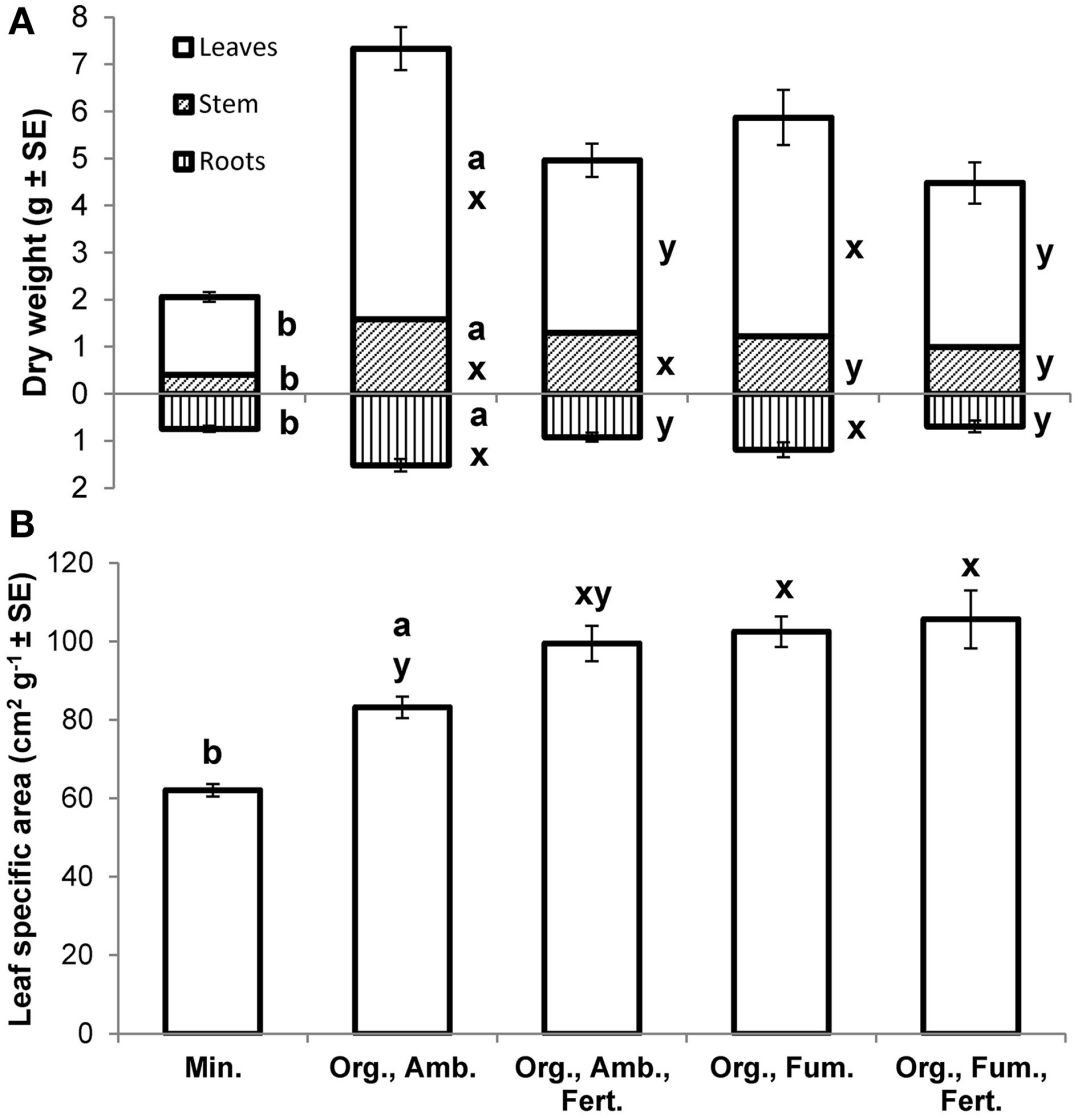
**Mean dry weights (g ± SE) of leaves, stems (including petioles), and roots (A) and mean leaf specific areas (cm^2^ g^−1^ ± SE; B) of *Eucalyptus obliqua* seedlings 357 days after transplant. Mean root dry weights are shown as positive values beneath the x-axis**. For each plant part separately, bars to the left of the same lowercase letter **(A)** or those topped by the same lowercase letter **(B)** do not differ significantly at *P* ≤ 0.05 by Welch's mean differences test for Min. vs. Org., Amb. (a,b), or by Tukey's honestly significant difference test among all four organic soil treatments (x,y). *n* = 17, 17, 16, 17, and 15 (leaves, stem, and leaf specific area) or 14 (roots), for the five treatments, respectively. Min., mineral soil; Org., organic soil; Amb., ambient soil (i.e., not fumigated); Fum., fumigated with methyl bromide gas; Fert., fertilized with chelated iron followed by phosphate (see Materials and Methods).

At the final harvest, *E. obliqua* seedlings in ambient organic soil had higher mean dry weights of leaves [*F*_(1, 17.7)_ = 114.07, *P* < 0.0001], stems [*F*_(1, 17.2)_ = 103.70, *P* < 0.0001], roots [*F*_(1, 23)_ = 27.85, *P* < 0.0001], and thus total plant [*F*_(1, 18.4)_ = 164.56, *P* < 0.0001] than those in mineral soil (Figure 5A), but their mean root-to-shoot ratios (ambient organic = 0.25; mineral = 0.38) did not differ [*F*_(1, 25.6)_ = 2.65, *P* = 0.1158]. Mean leaf specific areas [*F*_(1, 26.1)_ = 44.34, *P* < 0.0001] were higher for plants in ambient organic than in mineral soil (Figure 5B).

Among all organic soil treatment final harvest dry weight means, after Bonferroni correction fumigation significantly diminished only stem dry weight [*F*_(1, 61)_ = 8.29, *P* = 0.0055] while fertilization significantly diminished leaf [*F*_(1, 61)_ = 19.39, *P* < 0.0001], root [*F*_(1, 60)_ = 17.34, *P* < 0.0001], and total plant mean dry weight [*F*_(1, 60)_ = 20.61, *P* < 0.0001]. Neither treatment significantly affected root-to-shoot ratio [grand mean = 0.20; fumigation *F*_(1, 60)_ = 1.07, *P* = 0.3057; fertilization *F*_(1, 60)_ = 1.81, *P* = 0.1839], nor was any fumigation × fertilization interaction significant (all *P* ≥ 0.2154). Both fumigation [*F*_(1, 61)_ = 7.05, *P* = 0.0101] and fertilization [*F*_(1, 61)_ = 4.10, *P* = 0.0472], but not their interaction [*F*_(1, 61)_ = 1.88, *P* = 0.1750], increased leaf specific area (Figure 5B).

Leaf mineral nutrient concentrations of N, P, S, Ca, Mg, Na, Mn, and Zn were 1.6 (N) to 3.3 (Mn) times greater (Table 2) for plants in ambient organic than in mineral soil (all Welch's test *P* ≤ 0.0001 except Mg and Zn *P* ≤ 0.0002, and N *P* ≤ 0.0019). Only mean foliar iron concentration was 1.6 times higher for plants in mineral than in ambient organic soil [*F*_(1, 26.2)_ = 39.48, *P* < 0.0001]. The ratio of N to P (23.6 vs. 17.1) also was higher for plants in mineral than in ambient organic soil [*F*_(1, 22.6)_ = 21.67, *P* = 0.0001]. Plants in mineral soil had foliar concentrations of P, K, S, and Mn that were conspicuously lower than the ranges or values reported as “adequate” for youngest mature leaves of adult *E. obliqua* (Judd et al., 1996) and *E. regnans* (Reuter and Robinson, 1997). Only the foliar concentration of Cu was more than seven times the adequate range (Table 2). In consequence of the large difference in mean leaf dry weight between plants in mineral soil vs. ambient organic soil, total foliar contents of all twelve elements analyzed were significantly greatest (all *P* < 0.0001) for plants grown in ambient organic soil.

**Table 2:** **Mean foliar element concentrations (± SE) of *Eucalyptus obliqua* seedlings grown in mineral and organic (Org.) soils at the final harvest, 357 days after transplant, and adequate concentrations from healthy, mature trees shown for comparison**.

**Element Units**	**Mineral**	**Org., Amb.^a^**	**Org., Amb., Fert**.	**Org., Fum**.	**Org., Fum., Fert**.	**Adequate^b^**
Total nitrogen %	**0.69 b**^c^ (0.09)	**1.09 a x** (0.09)	1.13 x (0.09)	1.21 x (0.09)	0.96 x (0.10)	0.68–1.36
Phosphorous %	**0.03 b** (0.03)	**0.065 a y** (0.03)	**0.56 x** (0.03)	**0.069 y** (0.03)	**0.52 x** (0.03)	0.057–0.080
N:P	**23.6 a** (1.3)	**17.1 b x** (0.6)	**2.2 y** (0.2)	**16.7 x** (0.7)	**2.2 y** (0.3)	
Potassium %	0.21 a (0.02)	0.21 a x (0.02)	0.21 x (0.02)	0.24 x (0.02)	0.21 x (0.02)	0.32–0.45
Sulfur %	**0.07 b** (0.007)	**0.13 a x** (0.007)	**0.11 y** (0.007)	**0.13 x** (0.007)	**0.10 y** (0.007)	0.14
Calcium %	**0.53 b** (0.05)	**0.98 a x** (0.05)	0.89 x (0.05)	0.89 x (0.05)	0.78 x (0.05)	0.39–0.66
Magnesium %	**0.20 b** (0.02)	**0.33 a x** (0.02)	0.40 x (0.02)	0.37 x (0.02)	0.37 x (0.02)	0.24–0.39
Sodium %	**0.14 b** (0.02)	**0.27 a y** (0.02)	**0.48 x** (0.02)	**0.38 y** (0.02)	**0.51 x** (0.02)	0.15
Iron mg kg^−1^	**83.90 a** (12.84)	**52.24 b y** (12.84)	**55.09 x** (13.23)	**50.60 y** (12.84)	**119.54 x** (13.67)	72
Manganese mg kg^−1^	**328.4 b** (63.64)	**1081.2 a x** (63.64)	1031.8 x (65.60)	1066.6 x (63.64)	1267.9 x (67.75)	580–1200
Zinc mg kg^−1^	**11.88 b** (1.94)	**23.81 a x** (1.94)	**17.23 y** (2.00)	**32.07 x** (1.94)	**18.83 y** (2.06)	8–18
Copper mg kg^−1^	58.16 a (6.15)	64.28 a x (6.15)	53.84 x (6.34)	69.80 x (6.15)	53.69 x (6.55)	4–8
Boron mg kg^−1^	18.03 a (0.93)	18.04 a x (0.93)	17.65 x (0.96)	15.20 x (0.93)	17.83 x (0.99)	19

a *Amb., ambient soil (i.e., not fumigated); Fum., fumigated with methyl bromide gas; Fert., fertilized with chelated iron followed by phosphate (see Materials and Methods)*.

b *Adequate concentration ranges based upon youngest mature foliage from healthy trees; N, P, K, Ca, and Mg from E. obliqua in Table 17 of Judd et al. (1996); all other elements from E. regnans, pp. 533–534 in Reuter and Robinson (1997)*.

c *For each element or ratio, means accompanied by the same lowercase letter do not differ at P = 0.0019 by Welch's mean differences test for Min. vs. Org., Amb. (a,b), or at P = 0.0081 by Tukey's honestly significant difference test among all four organic soil treatments (x–z). Significantly different values are shown in bold. n = 17, 17, 17, 16, and 15 for the five treatments, respectively (except n = 16 for Org., Amb., Fert. for P, K, S, Ca, Mg, and Na)*.

In organic soil after Bonferroni correction, neither fumigation nor the fumigation × fertilization interaction significantly affected the mean concentrations of any element analyzed in *E. obliqua* foliage (Table S1). Fertilization, however, significantly affected P [*F*_(1,60)_ = 426.07, *P* < 0.0001], Na [*F*_(1,60)_ = 45.69, *P* < 0.0001], Zn [*F*_(1,61)_ = 20.39, *P* < 0.0001], and N:P [*F*_(1,60)_ = 895.57, *P* < 0.0001], and marginally significantly affected Fe [*F*_(1,61)_ = 8.22, *P* = 0.0057] and S [*F*_(1,60)_ = 7.50, *P* = 0.0081]. Fertilized plants had more P, Na, and Fe than non-fertilized plants, but had less Zn, S, and much lower N:P (2.2 vs. 16.9) than non-fertilized plants (Table 2). On average, plants in all organic soil treatments had about one-third the “adequate” K concentration, and those in all except fumigated, fertilized organic soil had just three-quarters the adequate Fe concentration. In contrast, plants in all organic soil treatments had at least 1.3, 2.7, and 7.6-fold the adequate Ca, Na, and Cu concentrations, respectively. Plants in fertilized organic soil had at least 4.5-fold the adequate P concentration, and those not fertilized had at least 1.6-fold the adequate Zn concentration (Table 2).

In consequence of the higher mean leaf dry weight in non-fertilized than in fertilized organic soil, even after Bonferroni correction, all elements analyzed except P, Fe, and Na had total foliar contents diminished by fertilization (all *P* ≤ 0.0008). The content of P was increased by fertilization [*F*_(1,60)_ = 473.69, *P* < 0.0001], but Fe [*F*_(1,61)_ = 0.00, *P* = 0.9611] and Na [*F*_(1,60)_ = 0.20, *P* = 0.6598] contents were not affected by fertilization. After Bonferroni correction, fumigation significantly affected only N content [*F*_(1,61)_ = 45.61, *P* = 0.0011] by diminishing it. There were no significant fumigation × fertilization interactions after Bonferroni correction for the foliar contents of any element analyzed (Table S1; all *P* ≥ 0.0242).

## Discussion

Although we hypothesized initially that *E. obliqua* seedlings would grow better in organic layer soil than in the mineral soil collected beneath it, our most surprising finding was how much better they grew. Whole plant dry weight was more than three times greater in ambient organic than in mineral soil at 357 DAT. As we also hypothesized, methyl-bromide fumigation of organic soil on average halved the percentage of ectomycorrhizal root tips at the first harvest, even though we sampled the largest plants most likely to have sustained mycorrhizas. Nevertheless, by the final harvest there were no differences in mean percentage ectomycorrhizal root tips, and fumigation significantly diminished only stem dry weight by 23%. Contrary to our third hypothesis, fertilization consistently had negative effects on final harvest mean dry weights, reducing whole plant dry weight by 31%. Examination of seedling growth over time, however, revealed that Fe fertilization diminished seedling growth while P supplementation had positive effects revealed by fertilized seedlings attaining morphological sizes similar to those of non-fertilized seedlings by the final harvest.

### Seedling growth in mineral vs. organic soil

The mineral soil in our experiment was a conspicuously poorer medium for plant growth than was ambient organic soil even though both soils had been air-dried before being dispensed to pots, which Ashton and Kelliher (1996) suggested may simulate the ashbed effect by increasing the fertility of mineral soil (but see Launonen et al., 2005). Field organic soil was many times richer than field mineral soil for all elements except Al and Fe, which were 3.8 and 2.8 times richer in field mineral than organic soil, respectively, and nitrate N which was uniformly very low. Ambient organic soil remained richer than mineral soil over the course of the experiment, although the differences between them diminished for all elements except nitrate N and Na, which did not change, and Fe and Cu for which the differences between mineral and ambient organic soils increased. Increased differences, consistent with proportionally less withdrawal from one soil than the other, suggest that Fe was sufficient in mineral soil and Cu was sufficient in organic soil. Foliar mineral nutrient concentrations generally reflected soil fertility differences such that for seedlings grown in mineral soil, P, K, S, and Mn concentrations were one-half to two-thirds those deemed adequate for adults (Judd et al., 1996; Reuter and Robinson, 1997), but other elements including Fe were adequate. Only Cu was more than seven times the maximum adequate concentration for adult *E. regnans* and more than twice the 3–24 mg kg^−1^ adequate range for juvenile trees of the distantly related *E. globulus* Labill. (Reuter and Robinson, 1997). Excessive Al and Cu both can inhibit root growth (Foy, 1992), but we did not see predominant alteration of fine root morphology suggestive of toxicity. Furthermore, plants grown in mineral soil had a similar mean Cu concentration to those grown in organic soil even though field mineral soil had just 22% of the DTPA-extractable Cu of field organic soil, thereby suggesting a possible high Cu requirement of *E. obliqua* seedlings. In addition, ectomycorrhizas were abundant, and some ectomycorrhizal fungi may ameliorate metal toxicity including that of Al and Cu (Meharg, 2003; Antoniolli et al., 2010; Targhetta et al., 2013).

Contrary to our expectation, Fe addition to organic soil did not improve seedling growth even though several plants in non-fertilized ambient and fumigated organic soil showed inter-vein chlorosis. Although *E. obliqua* seedlings from acid soils can display severe chlorosis on calcareous soil (Anderson and Ladiges, 1978) which can be remedied by Fe fertilization (Anderson, 1983), both our mineral and organic soils were acidic and our *E. obliqua* seeds were collected in the same vicinity as the soils. Moreover, even though Mn deficiency can cause foliar chlorosis similar to that reflecting Fe deficiency (Czerniakowski et al., 2006), organic soil had more extractable Mn than mineral soil, and foliar concentrations of Mn in all organic soil treatments were near the adequate maximum (Reuter and Robinson, 1997). Therefore, Mn was unlikely to have contributed to leaf chlorosis of seedlings in organic soil. Instead, chlorosis in organic soil might have been a consequence of less DTPA-extractable Fe than in mineral soil and retarded ectomycorrhiza formation after fumigation. In mineral soil, the less than adequate foliar Mn concentration might have reflected retarded root growth (Parsons and Uren, 2007) because of soil density, low pH, and relatively high exchangeable Al. In particular, with ten times the bulk density of the organic soil, our mineral soil drained poorly and its structure lacked the improvement that burning might have produced (Chambers and Attiwill, 1994; McIntosh et al., 2005). The bulk density of our mineral soil was close to that reported to cause 50% height and diameter growth reductions for *E. regnans* (Rab, 1996).

The unexpected negative effects of Fe fertilization on seedling growth in organic soil that we found most likely were caused by Fe-immobilization of P (Yuan and Lavkulich, 1994). Attiwill (1962) pointed out that the amount of available P in the surface soil of a mature *E. obliqua* stand is less than optimum for seedling growth, and our study confirms that for both the organic and mineral layers. In non-fertilized soils of our experiment, P was the element most likely to have limited *E. obliqua* seedling growth, even though leaf K and Fe concentrations were only two-thirds and three-quarters, respectively, those adequate for adults. Phosphorus limitation may have been most severe in mineral soil as suggested by mean seedling N:P of 23.6 for mineral soil vs. 17.1 in ambient organic soil. Generally, N:P ratios of 14–16 are thought to indicate N and P co-limitation of plant growth with higher values indicating progressively more pronounced P limitation (Koerselman and Meuleman, 1996). In accord, Cromer et al. (1981) suggested that the optimum ratio for *E. globulus* and *E. sieberi* L. A. S. Johnson was “around 15.” Foliar N concentrations in our seedlings grown in organic soil were similar to those reported for ca. 2.5 year-old *E. obliqua* by Millner and Kemp (2012), but P concentrations in non-fertilized organic soil, although within the adequate range for mature trees (Judd et al., 1996), were half those reported by Millner and Kemp (2012), while in fertilized organic soil, P concentrations were double those reported (Millner and Kemp, 2012). Most notably, our seedlings in mineral soil had only 17% the P concentration reported by Millner and Kemp (2012) and 53% of the lowest adequate concentration given for adults by Judd et al. (1996). Moreover, the lower leaf specific areas that we observed for seedlings in mineral vs. ambient organic soil are consistent with greater mineral nutrient limitation (Ordoñez et al., 2009), in mineral than in organic soil. Interestingly, however, mean root-to-shoot ratios did not differ significantly in mineral and organic soils in spite of greater mineral nutrient limitation in the former, perhaps because of abundant ectomycorrhizas in both soils.

### Fumigation, mycorrhizal colonization and seedling growth in organic soil

Our fumigation of organic soil might have mimicked some aspects of the ashbed effect (Bowman and Fensham, 1995), although we could not statistically test for effects of fumigation of organic soil in the absence of fertilization. Fumigation without fertilization appeared to elevate ammonium 27-fold, P 1.8-fold, Fe 1.5-fold, and Mn 2.0-fold. Chambers and Attiwill (1994) similarly found that autoclaving, gamma irradiation, and ethylene dioxide fumigation all elevated available N, and that autoclaving transiently elevated water-soluble Mn in an *E. regnans* forest soil. Nevertheless, during the first measurement interval of our experiment, fumigation had negative effects on *E. obliqua* seedling growth that persisted well into the second interval, suggesting that effects of fumigation retarding ectomycorrhiza formation predominated over any improvement of soil fertility by fumigation.

By the final harvest, the mean percentage ectomycorrhizal root tips as well as the predominant ectomycorrhiza morphotypes did not differ among organic soil treatments suggesting that fumigation may not have eliminated fungus propagules entirely, and that fertilization did not suppress mycorrhiza formation. Ashton (1976) and Launonen et al. (2005) similarly observed for *E. regnans* that phosphate fertilization did not diminish ectomycorrhiza formation (although high levels of nitrogenous fertilizer did). Despite abundant ectomycorrhizas in organic soil, seedling dry weights were not significantly positively associated with percentage ectomycorrhizas unless the seedlings had been fertilized, again attesting to suboptimal phosphorus availability in ambient organic soil (Attiwill, 1962). The mean percentage ectomycorrhizas was slightly higher in mineral than in organic soil, which might have been a consequence of limited root growth in mineral soil (e.g., Torti et al., 1997), but neither increased ectomycorrhizas nor a different, predominant fungus associate than in organic soil (as similarly found for *E. marginata* Donn ex Sm. by Reddell and Malajczuk, 1984) could compensate for the physical and chemical deficiencies of the mineral soil.

Our work leaves ambiguous whether *E. obliqua* can form arbuscular mycorrhizas. Anderson and Ladiges (1978) reported “endotrophic *Endogone*” mycorrhizas on *E. obliqua* seedlings grown in calcareous soils, with “endotrophic” formerly representing arbuscular mycorrhizas. Subsequently, however, they mentioned “fruiting sporocarps of an *Endogone*-like species” which were abundant on roots of seedlings from non-burnt soil in a “type of ectomycorrhizal association” (Anderson and Ladiges, 1982). Warcup similarly reported ectomycorrhizas formed by “endogonaceous fungi” including *Glomus tubiforme* Tandy [now *Densospora tubaeformis* (Tandy) (McGee, 1996)] in mixed *E. obliqua* and *E. regnans* forests in Tasmania (Warcup, 1991), and on *E. regnans* in pots (Warcup, 1985). Warcup (1991) did report “vesicular-arbuscular mycorrhizae” in three of ten mixed *E. obliqua* and *E. regnans* regeneration coupes, but not in the five coupes that exclusively contained *E. obliqua*.

Whether *E. obliqua* seedlings can form arbuscular mycorrhizas might influence seedling performance, especially in the presence of exclusively arbuscular mycorrhizal plant species (Janos et al., 2013). The benefits of arbuscular mycorrhizas to eucalypts usually are considerably smaller than those of ectomycorrhizas (Lapeyrie and Chilvers, 1985; Jones et al., 1998; Chen et al., 2000; Kariman et al., 2012). Moreover, when eucalypts are inoculated simultaneously with arbuscular and ectomycorrhizal fungi, ectomycorrhizal root colonization may be diminished vs. inoculation with ectomycorrhizal fungi alone (Kariman et al., 2012). At worst, arbuscular mycorrhizas may have direct detrimental effects on eucalypt seedling growth (Oliveira et al., 1995) including disadvantageously linking eucalypt seedlings to competing vegetation through common arbuscular mycorrhizal networks (Janos et al., 2013). In addition to being poor competitors when shaded (Barrett and Ash, 1992; Dignan et al., 1998; Van Der Meer et al., 1999; Alcorn, 2002), pioneer eucalypts also may be weak competitors belowground, depending on fire to eliminate other, predominantly arbuscular-mycorrhizal vegetation (Gilbert, 1959; Cunningham, 1960; Loneragan and Loneragan, 1964; Noble, 1980; Bowman and Kirkpatrick, 1984, 1986; Ashton, 1986; Wilkinson, 2008).

### Seedling growth in fertilized organic soil

As long-persistent, fire-pioneer species (Tng et al., 2012), giant eucalypts may have abdicated the ability to develop extensive root systems (and thereby, abdicated a large part of their belowground competitive ability) in favor of emphasizing aboveground growth because they must avoid being overtopped. When competing vegetation has been eliminated by fire, eucalypt seedings likely take up available mineral nutrients initially without any time-lag associated with awaiting mycorrhizal colonization (Janos, 1980) by depending upon root hairs (Ashton, 1976; Launonen et al., 2004). A variety of pyrophillic, ectomycorrhizal fungi (Warcup, 1991; McMullan-Fisher et al., 2011), however, can be depended upon to colonize seedling roots and probably to assist in mineral nutrient uptake, especially of P, shortly thereafter. High levels of available P after fire are not likely to suppress ectomycorrhizas (Loneragan and Loneragan, 1964; Ashton, 1976; Launonen et al., 2005). Thus, it was surprising that we found a significant negative effect of P fertilization on leaf length change in fumigated organic soil. Because those seedlings showed neither a significant reduction of ectomycorrhizas nor an increase of leaf specific area at 357 DAT, the most likely explanation for retarded leaf length increase might have been an increase in branching associated with fertilization. Loneragan and Loneragan (1964) similarly noted that fertilization increased branching by *E. diversicolor* F. Muell. While our P fertilization of organic soil did accelerate seedling growth, its most pronounced effect was to have shifted seedlings from likely P limitation (mean N:P = 16.9) to potential K or eventual N limitation (mean N:P = 2.2) consistent with Cromer et al. (1981) finding that N fertilization in the field increased the height of 5-year old *E. obliqua* saplings.

### Forestry management implications

Our results strongly question the notion prevalent in forest management (Neyland et al., 2009) that because of requisite fertility improvement of exposed mineral soil a burnt seedbed—an ashbed—is necessary to ensure adequate stocking of *E. obliqua* seedlings after timber harvest. Indeed, one dispersed-retention coupe (WR1B) studied by Neyland et al. (2009) achieved a commercially acceptable seedling density despite a very poor burn, i.e., only 14% of the area burnt to mineral soil or ashbed. Regeneration success was anticipated after aggregated-retention harvesting followed by “slow-burning,” which resulted in only 13% of the area burnt to an ashbed (which low percentage did not differ from “clearfell, burn and sow” silviculture) while burnt to litter predominated on 28% of the area (Scott et al., 2012). We suggest that much of fire's ashbed effect on mineral soil may be a “remedy” for a problem created by fire, i.e., loss of the relatively fertile organic soil layer. The most important contribution of fire actually may be “weed control” that temporarily frees eucalypt seedlings from both aboveground (Prior and Bowman, 2014) and belowground interspecific competition.

Our results suggest that *E. obliqua* seedlings might be able to establish successfully without fire in wet eucalypt forests if interspecific competition can be minimized while phosphorus availability is elevated. If woody debris could be removed and utilized (e.g., for vehicle fuel production, see http://www.topsoe.com/about_us/green_commitment/tigas.aspx), then control of competing vegetation regrowing as root sprouts or from the soil seedbed (Hindrum et al., 2012) by several bouts of repeated re-clearing prior to eucalypt sowing might be feasible. That could diminish belowground competition and disrupt arbuscular mycorrhizal networks (Janos et al., 2013) while allowing decomposition of non-woody debris to enrich the organic layer. Nevertheless, some fertilization likely would be needed after eucalypt sowing. Although the management decisions of commercial forestry ultimately will be based on safety to operational staff and economic return (Neyland et al., 2012), reduction of smoke and carbon dioxide release might elevate the potential of non-burn alternatives for *E. obliqua* forest management.

### Conflict of interest statement

The authors declare that the research was conducted in the absence of any commercial or financial relationships that could be construed as a potential conflict of interest.

## Supplementary material

Figure S1 **Mean height (mm ± SE; A) and mean number of leaves (±SE; B) vs. days after transplant of *Eucalyptus obliqua* seedlings**. Seedlings were transplanted to mineral soil (solid red line, filled squares; “Mineral”) or organic layer soil (blue lines; “Org.”) that was either fumigated (dashed line, open circles; “Fum.”) or not (solid line, filled circles; “Amb.”) and/or fertilized (dotted lines, filled triangles; “Amb., Fert.;” or dotted lines, open triangles; “Fum., Fert.”). The fertilization regime began with chelated iron, but was changed to phosphate 150 days after transplant. All first harvest plants were excluded.
