# Supplementary figures and images for: Eucalyptus obliqua seedling growth in organic vs. mineral soil horizons
Source: Front Plant Sci. 2015 Feb 20;6:97. doi: 10.3389/fpls.2015.00097 (PMC4335303; doi:10.3389/fpls.2015.00097)

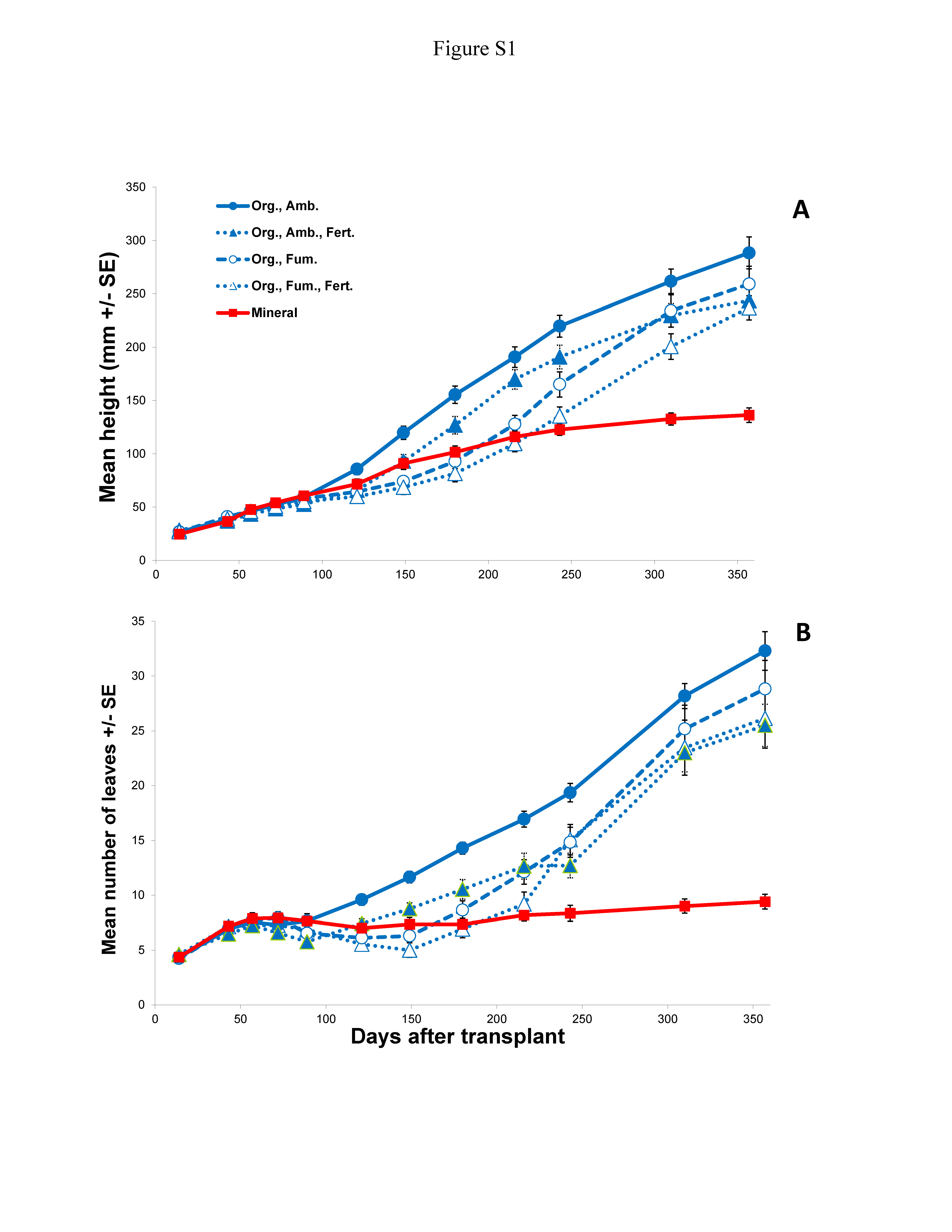

Supplement: Figure S1 — Mean height (mm ± SE; A) and mean number of leaves (±SE; B) vs. days after transplant of Eucalyptus obliqua seedlings. Seedlings were transplanted to mineral soil (solid red line, filled squares; “Mineral”) or organic layer soil (blue lines; “Org.”) that was either fumigated (dashed line, open circles; “Fum.”) or not (solid line, filled circles; “Amb.”) and/or fertilized (dotted lines, filled triangles; “Amb., Fert.;” or dotted lines, open triangles; “Fum., Fert.”). The fertilization regime began with chelated iron, but was changed to phosphate 150 days after transplant. All first harvest plants were excluded. [file Image1.JPEG]
