# Supplementary material for: Eucalyptus obliqua seedling growth in organic vs. mineral soil horizons
Source: Front Plant Sci. 2015 Feb 20;6:97. doi: 10.3389/fpls.2015.00097 (PMC4335303; doi:10.3389/fpls.2015.00097)
Supplement: Supplementary file 2 [file Table1.DOCX]

**Table S1. *F*-statistics and associated probabilities (shown parenthetically) of two-way, factorial analyses of variance for leaf tissue element concentrations (Conc.) and contents (Cont.) of *Eucalyptus obliqua* seedlings grown in ambient, fumigated (Fum.), or fertilized (Fert.) organic soil.**

|  |  | Conc. |  |  |  | Cont. |  |
| --- | --- | --- | --- | --- | --- | --- | --- |
| Element Error DF^1^ | Fum. *F* (*P*) | Fert. *F* (*P*) | Fum. × Fert. *F* (*P*) |  | Fum. *F* (*P*) | Fert. *F* (*P*) | Fum. × Fert. *F* (*P*) |
| Total nitrogen 61 | 0.06 (0.8082) | 1.16 (0.2851) | 2.11 (0.1518) |  | **11.67**^2^ **(0.0011)** | **45.61** **(< 0.0001)** | 0.08 (0.7835) |
| Phosphorous 60 | 0.28 (0.5985) | **426.07 (< 0.0001)** | 0.84 (0.3633) |  | 5.33 (0.0244) | **473.69** **(< 0.0001)** | 0.48 (0.4930) |
| N:P 60 | 0.11 (0.7446) | **895.57** **(< 0.0001)** | 0.17 (0.6847) |  |  |  |  |
| Potassium 60 | 1.04 (0.3113) | 0.67 (0.4154) | 0.84 (0.3642) |  | 0.68 (0.4127) | **59.63** **(< 0.0001)** | 0.01 (0.9290) |
| Sulphur 60 | 0.00 (0.9747) | 7.50 (0.0081) | 0.91 (0.3433) |  | 3.27 (0.0754) | **74.89** **(< 0.0001)** | 0.21 (0.6464) |
| Calcium 60 | 3.91 (0.0525) | 3.96 (0.0510) | 0.03 (0.8579) |  | 5.94 (0.0178) | **33.34** **(< 0.0001)** | 0.78 (0.3807) |
| Magnesium 60 | 0.03 (0.8698) | 2.71 (0.1049) | 3.51 (0.0660) |  | 0.63 (0.4302) | **12.37** **(0.0008)** | 0.48 (0.4922) |
| Sodium 60 | 7.00 (0.0104) | **45.69 (< 0.0001)** | 2.48 (0.1202) |  | 0.36 (0.5496) | 0.20 (0.6598) | 0.44 (0.5091) |
| Iron 61 | 2.20 (0.1427) | 8.22 (0.0057) | 5.48 (0.0225) |  | 0.03 (0.8698) | 0.00 (0.9611) | 5.35 (0.0242) |
| Manganese 61 | 2.33 (0.1323) | 1.09 (0.2997) | 2.98 (0.0893) |  | 0.14 (0.7121) | **15.14** **(0.0002)** | 2.92 (0.0925) |
| Zinc 61 | 5.05 (0.0283) | **20.39** **(< 0.0001)** | 2.30 (0.1342) |  | 0.07 (0.7926) | **13.57** **(0.0005)** | 0.09 (0.7597) |
| Copper 61 | 0.16 (0.6908) | 3.91 (0.0526) | 0.18 (0.6743) |  | 0.42 (0.5172) | **31.32** **(< 0.0001)** | 0.04 (0.8459) |
| Boron 61 | 2.07 (0.1549) | 1.48 (0.2279) | 2.71 (0.1051) |  | 4.53 (0.0374) | **13.76 (0.0005)** | 2.83 (0.0975) |

^1^ Error degrees of freedom. Main effects and interaction degrees of freedom = 1.

^2^ Effects that are significant after Bonferroni correction (*P* ≤ 0.0038 for concentration and N:P; *P* ≤ 0.0042 for content) are shown in bold.
